# Development and validation of a novel nomogram for pretreatment prediction of liver metastasis in pancreatic cancer

**DOI:** 10.1002/cam4.2930

**Published:** 2020-02-28

**Authors:** Shangxiang Chen, Shaojie Chen, Guoda Lian, Yaqing Li, Xijiu Ye, Jinmao Zou, Ruomeng Li, Ying Tan, Xuanna Li, Mengfei Zhang, Chunyu Huang, Chengzhi Huang, Qiubo Zhang, Kaihong Huang, Yinting Chen

**Affiliations:** ^1^ Department of Gastroenterology and the Guangdong Provincial Key Laboratory of Malignant Tumor Epigenetics and Gene Regulation Sun Yat‐sen Memorial Hospital Sun Yat‐sen University Guangzhou P.R. China; ^2^ Department of Endoscopy Sun Yat‐sen University Cancer Center State Key Laboratory of Oncology in South China Collaborative Innovation Center for Cancer Medicine Guangzhou P.R. China; ^3^ Department of General Surgery Guangdong General Hospital Guangzhou P.R. China; ^4^ Department of Anesthesiology Sun Yat‐sen Memorial Hospital Sun Yat‐sen University Guangzhou P.R. China; ^5^ Department of Gastroenterology Lihuili Hospital of Ningbo Medical Center Ningbo China

**Keywords:** diagnosis, liver metastasis, nomogram, pancreatic cancer

## Abstract

**Purpose:**

The diagnostic value of nomogram in pancreatic cancer (PC) with liver metastasis (PCLM) is still largely unknown. We sought to develop and validate a novel nomogram for the prediction of liver metastasis in patients with PC.

**Method:**

About 604 pathologically confirmed PC patients from the Sun Yat‐sen University Cancer Center (SYSUCC) between July, 2001 and December, 2013 were retrospectively studied. The SYSUCC cohort was randomly assigned to as the training set and internal validation set. Using these two sets, we derived and validated a prognostic model by using concordance index and calibration curves. Another two independent cohorts between August, 2002 and December, 2013 from the Sun Yat‐sen Memorial Hospital (SYSMH, n = 335) and Guangdong General Hospital (GDGH, n = 503) was used for external validation.

**Result:**

Computed tomography (CT) reported liver metastasis status, carcinoembryonic antigen (CEA) level and differentiation type were identified as risk factors for PCLM in the training set. The final diagnostic model demonstrated good calibration and discrimination with a concordance index of 0.97 and had a robust internal validation. The score ability to diagnose PCLM was further externally validated in SYSMH and GDGH with a concordance index of 0.93. The model showed better calibration and discrimination than CT, CEA and differentiation in each cohort.

**Conclusion:**

Based on a large multi‐institution database and on the routinely observed CT‐reported status, CEA level and tumor differentiation in clinical practice, we developed and validated a novel nomogram to predict PLCM.

## Graphical abstract

In this study, we first established and validated a novel model based on three clinical features (computer tomography, carcinoembryonic antigen and differentiated status) for liver metastasis in pancreatic cancer. As a novel complement of the conventional TNM staging system, this model exhibited improved discrimination compared with current methods for identifying the pancreatic ductal adenocarcinoma with liver metastasis. Moreover, we revealed that this model could be conveniently used to facilitate the pretreatment individualized prediction of pancreatic cancer with liver metastasis.

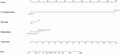

## 1 INTRODUCTION

Pancreatic ductal adenocarcinoma (PDAC) is among the most deadly cancers with a 5‐year survival rate of less than 6%,^1^ for its aggressive metastatic nature.^2^, ^3^ A preliminary analysis of 1620 PDAC cases from the Guangdong Province of China identified that 54.4% of the PDACs were diagnosed with distant metastasis, and more than half of which were liver metastases. Also, recent studies have reported that a large proportion of PDACs had liver metastasis at their initial diagnosis.^4^ As such, the accurate identification of liver metastasis is crucial for guiding strategic treatment decisions and prognostic assessments. Therefore, effective and easily accessible approach methods are urgently needed for the prediction and diagnosis of liver metastasis in PDACs patients.

Currently, histopathologic examination and imaging modalities such as computed tomography (CT) are the mostly commonly methods used to diagnose liver metastasis. However, the accuracy of preoperative histopathologic examinations is largely dependent on the quality of the punctured tissues. In addition, solely relying on CT scans has also been reported to be insufficient to accurately identify malignant lesions.^5^ To improve the sensitivity and specificity of each method, the advantages of these biomarkers (such as imaging features, histopathologic examination, and blood index values) should be incorporated. To analyze a panel of effective biomarkers as a group is the most promising method to change clinical management.^6^ To our knowledge, there is no literature reporting on a preoperative signature to improve the diagnosis of liver metastasis in PDAC.

Nomogram, comprehensively includes risk factors for prediction, has been found as a novel tool for such purpose. Recently, a nomogram for the prediction of lymph nodes metastasis in colorectal cancer has been established and validated^7^ and we hypothesize that such a strategy could identify liver metastasis in PDAC more accurately and effectively as compared to the current methods in practice. Thus, we aimed to construct and validate a nomogram to predict liver metastasis in PDAC patients.

## 2 PATIENTS AND METHODS

### 2.1 Patients selection

From July, 2001 to December, 2013, we retrospectively analyzed 604 patients (Primary dataset) who were hospitalized at the Sun Yat‐sen University Cancer Center (SYSUCC), Guangzhou, China. The patients included were histologically proven PDAC or PDAC with liver metastasis by preoperative biopsy, intraoperative exploration, or operative resection.

Additionally, from August, 2002 to December, 2013, another two independent datasets from the Sun Yat‐sen Memorial Hospital (SYSMH, n = 335) and the Guangdong General Hospital (GDGH, n = 503), which met the aforementioned criteria were also analyzed for external validation. Ethical approval for this retrospective analysis was obtained from the ethical committee of SYSMH.

### 2.2 Data collection

Patient and tumor related variables such as host status (ie age, gender), primary tumor characteristics (ie site, differentiation, carcinoembryonic antigen (CEA) level, carbohydrate antigen 19‐9 (CA19‐9) level, alpha‐fetoprotein (AFP) level, CT‐reported liver metastasis), and follow‐up data were reviewed. The level of CEA, CA19‐9, and AFP was obtained via laboratory analysis of the patients’ routine blood test at initial diagnosis, and the cutoff value was determined by the Youden‐index method.^8^ The tumor site was defined as head, body, tail, and overlapping lesions based on the location of the center of the lesion. As for the tumor differentiation type, well and moderately differentiated type were defined as the differentiated type, while the poorly differentiated were defined as the undifferentiated type. The CT diagnosis of pancreatic cancer (PC) with liver metastasis (PCLM) was performed by at least two radiologists to avoid the bias.

### 2.3 Constructing nomogram

The Primary dataset was randomly divided by computer‐aid into two groups, namely the Training and Internal validation dataset. Selection bias regarding the factors for the random classification into the two groups was adjusted.^9^ Lasso Cox regression analysis was used in the training set to identify the independent risk factors, based on which the nomogram was constructed.

### 2.4 Validating nomogram

Discrimination and calibration analysis were performed to evaluate performance of the nomogram in the training (SYSUCC, n = 302) and two other independent datasets (SYSMH, n = 335 and GDGH, n = 503). The Harrell's C‐index was used for the discrimination analysis.^10^, ^11^ The C‐index provides the probability between the observed and predicted probability of PDAC with liver metastasis. The C‐index could work as a measure of the accuracy of a nomogram.^12^ For the calibration of the nomogram, data were grouped based on the probabilities calculated by the nomogram predictive model. The predicted probabilities produced was then compared with the actual probabilities. H‐L chi‐square statistic and bootstrapping correction were used.^13^

Analysis were completed using the software statistical package for social sciences version 20.0 (SPSS, Chicago, IL) and the package of *glmnet* in R software version 3.5.1 (http://www.r-project.org/). *P* < .05 was considered as statistically significant.

### 2.5 Clinical applicability of the nomogram

To assess the clinical usefulness of the nomogram, decision curve analysis was performed. The net benefits at different threshold probabilities were quantified as per previously described.^14^

## 3 RESULTS

### 3.1 Patient demographics and outcomes

The data of the PCLM patients from the training (n = 302) and internal validation set (n = 302) were analyzed and no selective bias or significant difference in the investigated features between both groups was found (all *P* ＞ .05). The clinicopathologic features for the training and internal validation group are showed as Table 1. There were 129 and 130 patients who were ≥61 years old in training set and validation set separately. About 204 male in the training set and 200 male in the validation set. There were 110 and 104 PDAC with liver metastasis in the training and validation set, respectively, and the baseline clinical features for the two external validations are presented in Table 2.

**Table 1:** Clinical features of primary training set and validation set

	Training set (n = 302)		Validation set (n = 302)		*P*
	LM (+)	LM (−)	LM (+)	LM (−)	
Age (y)					
＜61	61 (55.5)	112 (58.3)	56 (53.8)	116 (58.6)	.934
≥61	49 (44.5)	80 (41.7)	48 (46.2)	82 (41.4)	
Gender					
Male	74 (67.3)	130 (67.7)	69 (66.3)	131 (66.2)	.729
Female	36 (32.7)	62 (32.3)	35 (33.7)	67 (33.8)	
Primary site					
Head	50 (45.5)	129 (67.2)	43 (41.3)	129 (65.2)	.133
Body	7 (6.4)	16 (8.3)	16 (15.4)	23 (11.6)	
Tail	22 (20.0)	12 (6.3)	22 (21.2)	14 (7.1)	
Overlapping lesions	31 (28.1)	35 (18.2)	23 (22.1)	32 (16.1)	
Differentiation					
Differentiated	18 (16.4)	95 (49.5)	23 (22.1)	89 (44.9)	.933
Undifferentiated	92 (83.6)	97 (50.5)	81 (77.9)	109 (55.1)	
CEA level (ng/mL)					
＜4.5	33 (30.0)	83 (43.2)	40 (38.5)	88 (44.4)	.098
≥4.5	77 (70.0)	69 (35.9)	64 (61.5)	86 (43.4)	
Unknown		40 (20.9)		24 (12.2)	
CA19‐9 level (ng/mL)					
＜386.6	40 (36.4)	67 (34.9)	36 (34.6)	77 (88.9)	.432
≥385.6	63 (57.3)	74 (38.5)	57 (54.8)	86 (43.4)	
Unknown	7 (6.3)	51 (26.6)	11 (10.6)	35 (17.7)	
AFP level (ng/mL)					
＜2.84	36 (32.7)	39 (20.3)	31 (29.8)	48 (24.3)	.374
≥2.84	28 (25.5)	35 (18.2)	28 (26.9)	47 (23.7)	
Unknown	46 (41.8)	118 (61.5)	45 (43.3)	103 (52.0)	
CT‐reported LM status					
Positive	93 (84.5)	5 (2.6)	87 (83.7)	2 (1.0)	.428
Negative	17 (15.5)	187 (97.4)	17 (16.3)	196 (99.0)	

Abbreviations: AFP, alpha fetoprotein; CA19‐9, carbohydrate antigen 19‐9; CEA, carcinoembryonic antigen; LM, liver metastasis.

**Table 2:** Clinical features of SYSMH set and GDGH set

	SYSMH set (n = 335)		GDGH set (n = 503)	
	LM (+)	LM (−)	LM (+)	LM (−)
Age (y)				
＜61	13 (48.1)	185 (55.1)	68 (38.9)	121 (36.9)
≥61	14 (51.9)	151 (44.9)	107 (61.1)	207 (63.1)
Gender				
Male	12 (44.4)	229 (68.2)	115 (65.7)	195 (40.5)
Female	15 (55.6)	107 (31.8)	60 (34.3)	133 (59.5)
Primary site				
Head	11 (40.7)	40 (11.9)	75 (42.9)	210 (64.0)
Body	3 (11.1)	88 (26.2)	19 (10.9)	26 (7.9)
Tail	5 (18.5)	71 (21.1)	38 (21.7)	29 (8.8)
Overlapping lesions	8 (29.6)	137 (40.8)	43 (24.6)	63 (19.2)
Differentiation				
Differentiated	7 (25.9)	15 (4.5)	43 (24.6)	104 (31.7)
Undifferentiated	20 (74.1)	321 (95.5)	132 (75.4)	224 (68.3)
CEA level (ng/mL)				
＜4.5	11 (40.7)	83 (24.7)	55 (31.4)	179 (54.6)
≥4.5	14 (51.9)	33 (9.8)	113 (64.6)	121 (36.9)
Unknown	2 (7.4)	220 (65.5)		28 (8.5)
CA19‐9 level (ng/mL)				
＜386.6	14 (51.9)	86 (25.6)	63 (36.0)	173 (52.7)
≥385.6	11 (40.7)	30 (8.9)	101 (57.3)	134 (40.9)
Unknown	2 (7.4)	220 (65.5)	11 (6.3)	21 (6.4)
AFP level (ng/mL)				
＜2.84	16 (59.3)	68 (20.2)	67 (38.3)	130 (39.6)
≥2.84	9 (33.3)	39 (11.6)	94 (53.7)	156 (47.6)
Unknown	2 (7.4)	229 (68.2)	14 (8.0)	42 (12.8)
CT‐reported LM status				
Positive	25 (92.6)	5 (2.6)	150 (85.7)	3 (0.9)
Negative	2 (7.4)	184 (97.4)	25 (14.3)	325 (99.1)

Abbreviations: AFP, alpha fetoprotein; CA19‐9, carbohydrate antigen 19‐9; CEA, carcinoembryonic antigen; LM, liver metastasis.

### 3.2 Risk factors screening

**Figure 1:**
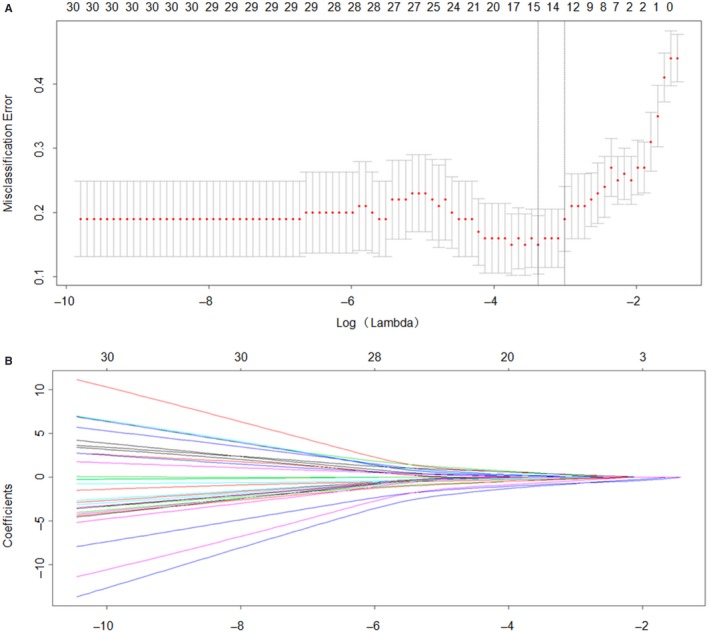
Clinicopathological features selection using the least absolute shrinkage and selection operator (LASSO) binary logistic regression model. A, Tuning parameter (λ) selection in the LASSO model used a 10‐fold cross‐validation via minimum criteria. The dotted vertical lines were drawn at the optimal values by using the minimum criteria and the 1 standard error of the minimum criteria (the 1‐SE criteria). B, Illustrate the LASSO coefficient profiles of the pancreatic cancer with liver metastasis‐associated clinical features. A coefficient profile plot was produced against the log (λ) sequence. A vertical line was drawn at the value selected chosen by 10‐fold cross‐validation

Factors were transformed and examined to fit the Logistic Regression.^9^, ^15^ Lasso Cox Regression was used to select the most useful clinical features for the diagnosis of PCLM in the training set. CEA level, tumor differentiation type and CT‐reported LM status were identified as the independent risk factors for PDAC with liver metastasis (Figure 1).

### 3.3 Construction and validation of the nomogram

**Figure 2:**
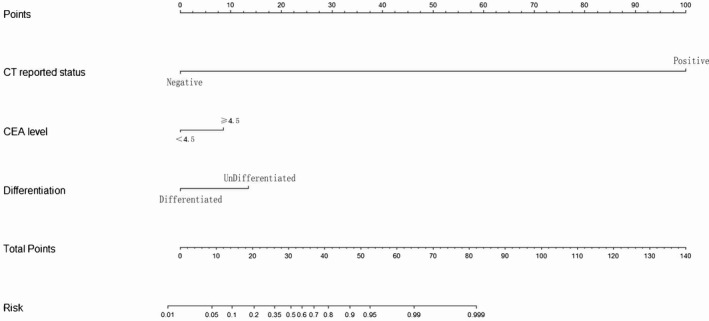
Developed pancreatic cancer with liver metastasis‐diagnostic nomogram. The nomogram was constructed using the training set. Routine clinicopathological feautures such as computer tomography‐reported liver metastasis status, carcinoembryonic antigen level and tumor differentiation type were identified as independent risk factors for pancreatic ductal adenocarcinoma with liver metastasis and were incorporated to build the nomogram

A nomogram comprising of the CEA level, tumor differentiation type and CT‐reported LM status was constructed based on the independent risk factors identified in training set. As illustrated in Figure 2, by adding up the points identified on the points scale, the nomogram can provide the risk for a PDAC patient to be diagnosed with liver metastasis.^16^ The C‐index for this nomogram (0.970) was superior to that of CT estimation alone (0.930), tumor differentiation type (0.680), and CEA level (0.670). Similar results were obtain upon analysis in the internal validation dataset (C‐index: nomogram, 0.930; CT estimation, 0.920; tumor differentiation type, 0.620; CEA level, 0.540) and the primary dataset (C‐index: nomogram, 0.940; CT estimation, 0.920; tumor differentiation type, 0.630; CEA level, 0.580). The C‐index for the model (0.930) also exhibited superior to the CT (0.920), CEA level (0.480), and differentiation (0.550) in the validation set. The nomogram has the optimal C‐index value in the primary set as well (0.940).

**Figure 3:**
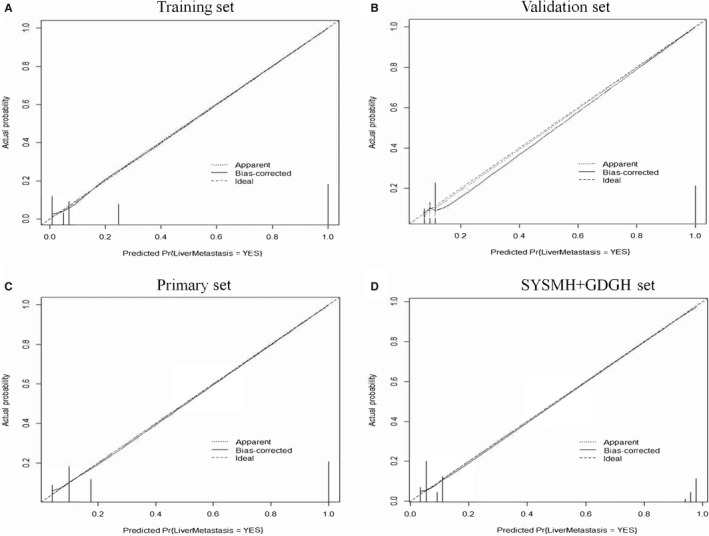
Illustrate the calibration curves of the liver metastasis (LM) predicting nomogram using the computer tomography‐reported LM status, carcinoembryonic antigen level and tumor differentiation type in the different dataset. A, Calibration curve of the diagnostic nomogram in the training set; B, Calibration curve of the diagnostic nomogram in the validation set; C, Calibration curve of the diagnostic nomogram in the primary set; D, Calibration curve of the diagnostic nomogram in the SYSMH + GDGH set. Calibration curves depict the calibration of each model in terms of the agreement between the predicted risks of pancreatic cancer with liver metastasis (PCLM) and observed outcomes of LM metastasis. The Y‐axis represents the actual PCLM rate. The X‐axis represents the predicted LM metastasis risk. The dotted line represents the ideal correlationship between predicted and actual survival

Further, the calibration of the three different datasets mentioned above was then performed. As illustrated in Figure 3, the apparent line was very close to that of the ideal line of liver metastasis, demonstrating reliable calibration for predicting the probability of PDAC with liver metastasis.

In the SYSM and GDGH external validation set, the C‐index of the nomogram (0.934) was also superior to that of the CT estimation (0.923), tumor differentiation type (0. 513), and CEA level (0. 644). Subsequently, the PDAC LM‐predictive nomogram maintained an optimal calibration and discrimination, as presented in (Figure 3D).

**Figure 4:**
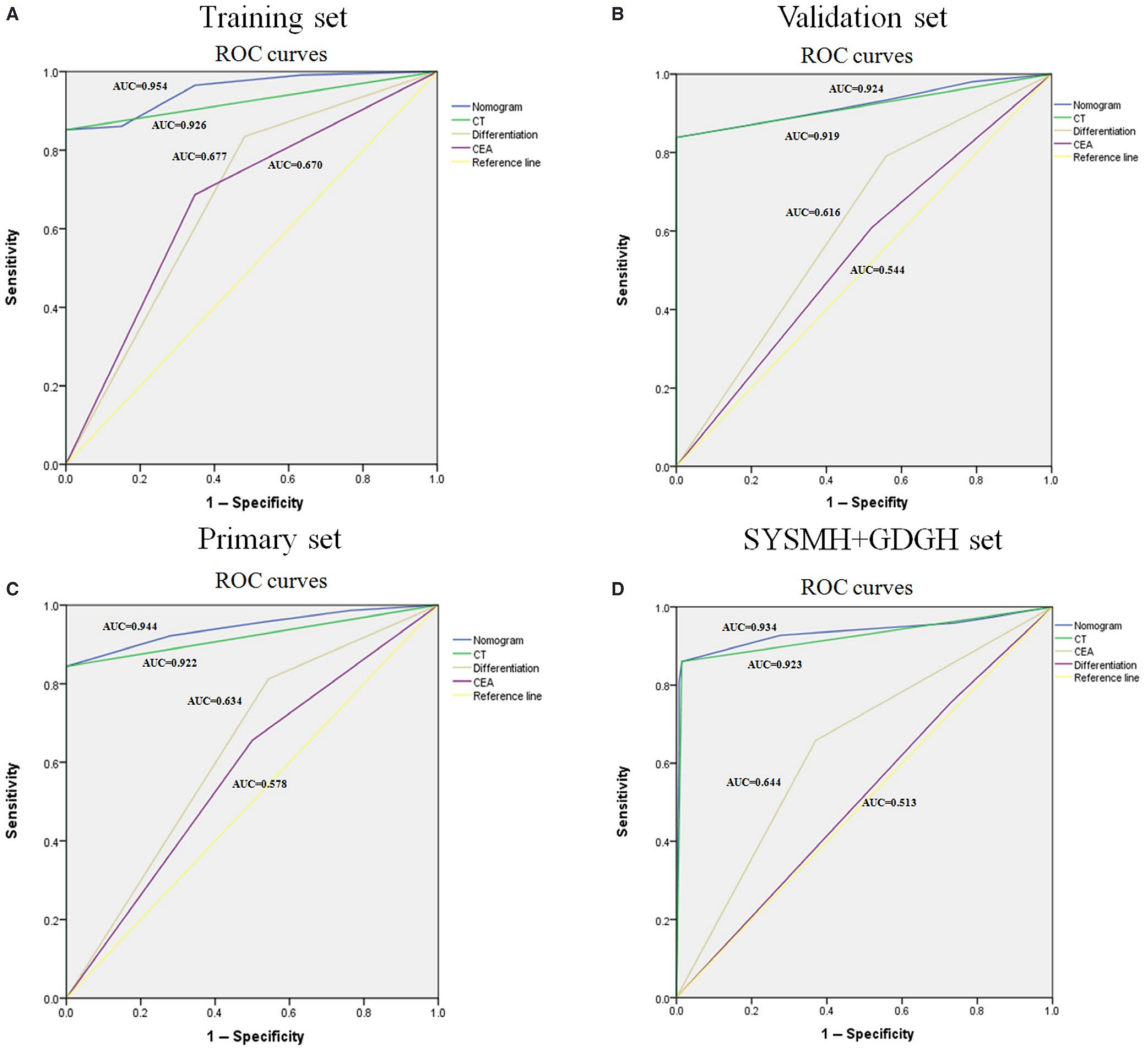
The ROC curves and AUCs values in the (A) training, (B) validation, (C) primary, and (D) SYSMH + GDGH sets

Further, as an estimation for the clinical reliability and practicability of this nomogram, we compared the efficiency between the nomogram and the individual variables by ROC curves. The nomogram consistently demonstrated the largest AUC value both in the training set (0.954), the internal validation set (0.924), and also upon external validation (0.934) (Figure 4).

### 3.4 Clinical use

**Figure 5:**
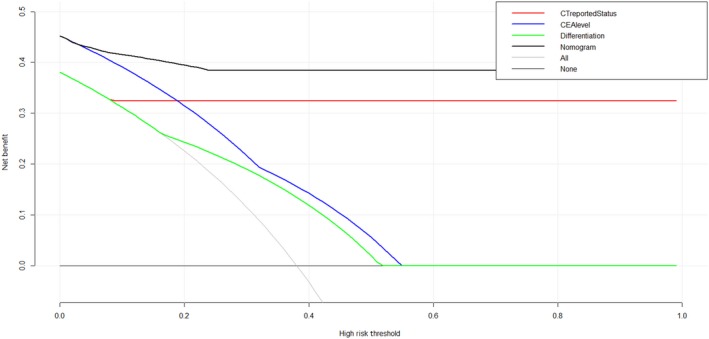
Decision curve analysis for the diagnostic nomogram and the individual clinical features. The Y‐axis measures the net benefit. The black line represents the diagnostic nomogram. The dark black line represents the diagnostic nomogram. The green line represents the tumor differentiation type. The blue line represents the carcinoembryonic antigen level. The red line represents the computer tomography‐reported liver metastasis (LM) status. The thin black line represents the assumption that all patients have LM metastases. Middle black line represents the assumption that no patients have LM metastases. The net benefit was calculated by subtracting the proportion of all patients who are false positive from the proportion who are true positive, weighting by the relative harm of forgoing treatment compared with the negative consequences of an unnecessary treatment

The decision curve analysis for our nomogram and the individual factors (CT, CEA level, and differentiation) are presented in Figure 5. As shown in figure, if the threshold probability of a patient or doctor is ＞10%, our nomogram adds more benefit to predict PCLM than all the individual factors.

## 4 DISCUSSION

In present study, using a large multi‐center population, we have developed and validated a pretreatment risk estimation nomogram for the prediction of liver metastasis in patients with PDAC. Three basic and routinely used clinicopathological features, namely the use of radiographic CT for the estimation of liver metastasis, CEA level and the tumor differentiation type were observed to be independent factors in the training set and were thus incorporated into the nomogram. This risk estimation nomogram demonstrated reliable and consistent results upon both internal and external validation. As such, this novel nomogram‐based model could stratify PDCA patients by predicting their risk of liver metastasis with high accuracy and may provide a more accurate approach for guiding individualized pretreatment therapeutic decisions.

CEA has been reported to have a low sensitivity of 39.5%, but acceptable specificity of 81.3% as a biomarker in PC.^17^ A previous study has showed that the CEA level was a reliable prognostic predictor in PDAC patients.^18^ Elevated levels of CEA were associated with poor prognosis for patients with PC.^19^, ^20^ However, until now, there have been no literature revealing the importance of the status of CEA level in the diagnosis of PCLM. The predictive value of the use of radiographic imaging via CT scans and tumor differentiation type in the diagnosis of PCLM were also not well described. Since these are clinically important and reliable methods routinely used in clinical practice to help diagnosis of PCLM, their combined use to build this risk‐predictive nomogram makes it clinically practical to serve as a more reliable approach for the diagnosis of PCLM.

Nomogram has been reported to be a novel tool for survival prediction in PC previously.^21^, ^22^, ^23^ However, it has not been applied in the diagnosis of PCLM yet. To construct a nomogram for the diagnosis of PCLM, a panel of features were incorporated into an integrated model. Actually, analysis of combined individual markers could improve the discrimination and has been widely used in recent studies.^6^ Panels of genes were identified and analyzed for their use in survival prediction.^24^, ^25^, ^26^ Similarly, the constructed nomogram incorporated multiple individual markers and has adequate discrimination in the training set, which was then demonstrated with good calibration and discrimination in the validation set as well. As for the comparable positivity of liver metastasis in the training and validation set, the improved calibration and discrimination indicated that the nomogram was extremely stable in the prediction of PCLM and could be used in validation set without adjusting the intercept and regression coefficients regarding the model building. Further validation was performed by two independent external validation set, which ascertained its wide application.

To validate the applicability of our model, we established and validated (by both internal and external validation) our nomogram by using a large cohort of multi‐institutional data. It is unreliable to assess a nomogram just by internal validation due to the data heterogeneity. External validation could be a complement to problems mentioned above. To justify its clinical usefulness, our nomogram was validated independently in SYSM and GDGH set to avoid selective bias and identify its universal applicability.^27^ Surprisingly, our nomogram showed satisfactory predictive value not only in training and internal validation set, but also in the external validation set. The comprehensive validations further ascertained the applicability of our model in different populations.

The most important and attracted point is the clinical use of the diagnostic model. We used the Lasso Cox Regression method to select the most useful markers of all the PCLM‐associated clinical factors. The method could both select predictors on the bias of the strength of their univariable association with clinical outcome, and combine the selective predictors into an integrated model.^28^, ^29^ Net benefit was also derived by the decision curve analysis method in this study, which offers us insight into the clinical benefit on the bias of threshold probability. In fact, if the threshold probability of a PDAC patient or doctor is ＞10%, using the diagnostic model in predicting PCLM adds more benefit. What is more, our model identified the definite risk factors of PCLM. Clinical features such as CT, CEA level, and differentiation type were first been revealed to be associated with the occurrence of PCLM in this study. Doctors and PDAC patients should pay more attention to these high‐risk factors before therapy decision. In addition, we first incorporated these valuable variables and built a nomogram. Both doctors and patients could perform an individualized pretreatment evaluation of the risk of PCLM with this easy to use scoring system, which may do great help in guiding personalized treatment.^30^

Certainly, there are still some limitations. First, this study analyzed PDAC patients from China. Whether this model will be suitable for other populations is yet to be demonstrated. Second, this study was a retrospective study, a prospective with larger population is required to further validate the results obtained.

In summary, based on a large cohort of patients, we propose a risk estimation nomogram which has demonstrated high accuracy, in both internal and external multi‐institution validation, for stratifying PDAC patients according to their probability of having PCLM based on three routinely used clinical features. In addition, this nomogram can be conveniently used in clinical practice to guide the pretreatment therapeutic selection for PDAC patients.

## CONFLICT OF INTEREST

All authors declare no conflicts of interest.

## AUTHOR CONTRIBUTIONS

Substantial contributions to the conception and design (Kaihong Huang, Yinting Chen), development of methodology (Shangxiang Chen, Shaojie Chen, Guoda Lian), acquisition of data (Yaqing Li, Xijiu Ye, Jinmao Zou, Ruomeng Li, Ying Tan, Xuannna Li, Mengfei Zhang, Chunyu Huang, Chengzhi Huang, Qiubo Zhang), analysis and interpretation of data (Shangxiang Chen), draft of the article (Shangxiang Chen, Kaihong Huang, Yinting Chen), critical revision of the article (all authors), and final approval of the version (all authors).

## Data Availability

The data of this study are available upon special request to the corresponding author(s).
